# Supplementary material for: The ‘fragile’ generation: Understanding parents’ attitudes towards young people’s mental health within the Chinese sociocultural context
Source: PLOS Ment Health. 2025 Jul 10;2(7):e0000340. doi: 10.1371/journal.pmen.0000340 (PMC12798485; doi:10.1371/journal.pmen.0000340)
Supplement: Attachment — Submitted filename: Response to Reviewers .docx [file pmen.0000340.s002.docx]

Response to Editor:

1. Please provide separate figure files in .tif or .eps format.

RESPONSE: We have now uploaded separate figure files in the required format.

2. Please provide an Author Summary. This should appear in your manuscript between the Abstract (if applicable) and the Introduction, and should be 150–200 words long. The aim should be to make your findings accessible to a wide audience that includes both scientists and non-scientists.

RESPONSE: We have added an Author Summary to the manuscript, which we hope will meet the criteria.

3. In the online submission form, you indicated that "Data will be made available on request.".

All PLOS journals now require all data underlying the findings described in their manuscript to be freely available to other researchers, either  a. In a public repository,  b. Within the manuscript itself, or  c. Uploaded as supplementary information.

This policy applies to all data except where public deposition would breach compliance with the protocol approved by your research ethics board. If your data cannot be made publicly available for ethical or legal reasons (e.g., public availability would compromise patient privacy), please explain your reasons by return email and your exemption request will be escalated to the editor for approval. Your exemption request will be handled independently and will not hold up the peer review process, but will need to be resolved should your manuscript be accepted for publication. One of the Editorial team will then be in touch if there are any issues.

RESPONSE: We apologize for the confusion regarding data provision requests. However, this study draws from qualitative data which contains potentially identifiable and sensitive information, as participants were invited to discuss their children’s age, gender, mental health difficulties, and personal life stories (which may have included 3^rd^ party identifying information). According to the information sheets and consent forms agreed by the participants (included in the Survey Questionnaire uploaded in the “Other” section), as well as the ethics protocol (see the Ethics Form uploaded in the “Other” section), the data cannot be shared with any third party or made publicly available.

Specifically, participants consented to the following condition: “Only my supervisor and I will have access to the data, which will be kept until September 2023 when the project is complete (see Page 2).” Furthermore, the ethics protocol states: “All data will be destroyed when the project ends by September 2023 (see Page 8).”

The project is not yet considered completed given the findings have not been published yet (i.e., writing and editing processes are still ongoing, and access to the data may be required in responding to peer reviews of the manuscript). Thus, the data have not been destroyed. They will be securely deleted following the publication of the study, which will mark its official completion.

Therefore, due to these ethical restrictions, we are unable to share the data once the manuscript has been published. If there are any questions about these ethical restrictions on public access to the data, they can be directed to: [ioe.researchethics@ucl.ac.uk](mailto:ioe.researchethics@ucl.ac.uk).

We have now removed the claim that "Data will be made available on request." from the manuscript to prevent any future confusion. The Data Availability Statement in our submission has also been updated accordingly

Responses to Reviewer 1:

Review Comments to the Author

Please use the space provided to explain your answers to the questions above. You may also include additional comments for the author, including concerns about dual publication, research ethics, or publication ethics. (Please upload your review as an attachment if it exceeds 20,000 characters)

Reviewer #1: Thanks for providing me the opportunity to review the manuscript. The main concerns are:

1. Some information might mislead international audience. For example, the authors pointed out that "currently a lack of official data about young people's mental health from Chinese institutions". In fact, there is an official dataset but it is not free for the public.

RESPONSE: We appreciate the Reviewer’s reminding us of this issue. Unfortunately, we do not have access to the official dataset because we do not work with institutions which are allowed access. We have rephrased this sentence to indicate that at the time of our writing no publically available official nationally representative dataset exists.

2. Although the authors emphasized that sociocultural might be an important influential factor on young people’s mental health, I do not think that they illustrate what a Confucianist family means clearly. The authors missed many key references in this section.

RESPONSE: We have revised the section introducing the Confucianist family in China, replacing some references with additional ones. As we intend to focus on how Confucianism might affect mental health care decisions specifically, we did not provide expansive explanations about Confucian families (in line with Reviewer 2’s suggestion to streamline the general information in the introduction). Instead, the introduction placed more attention on aspects of Confucian families that were central to the current research aims, namely the interactions between certain Confucianist values, such as hierarchal family relationships between children and parents, and decision-making in Chinese families.

3. Any ethics approval?

RESPONSE: As noted in the manuscript, this study was approved by the UCL Institute of Education Research Ethics Committee (IOE REC) on January 16, 2023. This article is based on data collected within the first author’s dissertation research, and therefore ethical review and approval was obtained via the dissertation ethics review procedures specified by the Institute of Education. This does not require full ethics committee review, and instead relies on an expedited review process provided by two independent academics in the department (i.e., the student’s supervisor and the Departmental Ethics Lead). Given the nature of this research involved potentially sensitive topics (i.e., children’s mental health), additional review was provided by a third independent member of the faculty, and approval was communicated via email on January 16^th^ 2023. This issue has been resolved with editors during previous communication through email (Approved by Jan Louis Adajo Non by email on 23/10/2024).

4. The recruitment process is not clear and the authors did not state clearly the rationale for using the method.

RESPONSE: The recruitment was conducted through purposive sampling online and snowball sampling. Invitation links to the survey were posted on popular social media platforms in China, including WeChat and Little Redbook. Participants were also kindly asked to invite others they knew who might have been interested in this study to participate by sharing a link to the study. An online survey was chosen over interviews because 1). It allowed for a larger sample size and diversified answers, which we believe to be important considering that this is the first study investigating this topic in post-pandemic time; 2). It may be more ethical because talking about their children’s mental health difficulties might be sensitive, and sometimes even shameful to some parents. A survey allows them to answer the questions without the presence of an outsider like the researcher being fully anonymous. We hoped that this could help participants feel safer and more open when answering the questions. We have added the rationale to the Method section on pages 9-10.

5. No questionnaire was provided. Personally, I do not know how the data was analyzed.

RESPONSE: We have uploaded the questionnaire to the “Other” section. It should now be accessible in the online supplemental materials.

6. Personally, I would suggest that the authors do a proof-reading again as many sentences are awkward.

RESPONSE: We have revised the manuscript and addressed the grammatical errors in the previous version of the manuscript. We hope that the Reviewers will find that the quality of the writing has improved.

Responses to Reviewer 2:

Reviewer #2: The manuscript provides an insightful qualitative exploration of Chinese parents' attitudes towards young people's mental health, contextualized within sociocultural influences such as Confucianism, the one-child policy, and stigma. The thematic analysis is comprehensive, and the authors effectively integrate relevant theoretical frameworks like labelling theory and Bronfenbrenner's ecological systems model. While this study is timely and topic, several concerns in writing and methodologies are worthy to be addressed before publication:

1. Introduction section. While the background is informative, the introduction could benefit from a more concise summary of the existing literature, focusing on gaps that this study aims to address, such as incorporating literature involving parental coping strategies and generational shifts. Currently, it feels somewhat repetitive and shallow.

RESPONSE: We have followed the helpful advice of the reviewer and consolidated the introduction, which is now one page shorter (including the new additional information on the Confucianist Family requested by Reviewer 1 in point 2). In addition to this consolidation, we have restructured the introduction to more clearly focus on the gaps that the current study aimed to address. We have tried to achieve this by renaming the subsections with key questions and introducing the figure earlier in the manuscript as a guide for the reader in following the sociocultural processes on which the study focused. Furthermore, the context in each subsection has been consolidated and leads into a final section which more clearly highlights the knowledge gaps and research questions guiding the current study.

2. Research Questions. The research questions are relevant, but they could be more sharply defined. For instance, RQ3 ("How do Chinese parents cope with young people's mental health-related problems?") could specify whether the focus is on practical strategies, emotional responses, or both. The logic to bridge background to research question should be smoothed in the Introduction section.

RESPONSE: We appreciate this suggestion and we have refined the research questions (Page 8) to show that the focus is on both practical strategies and emotional responses. As noted in our response to the Introduction section, we hope that the flow between the introduction and research question now reads more smoothly.

3. More concerns occur into the methodologies. One of the biggest methodological concerns is the under-representation in the sample diversity. The sample is heavily urban (96% urban residents). This limits the generalizability of the findings. Despite it has been acknowledged in the Limitation section, this point should be discussed in more detailed.

RESPONSE: Thank you for pointing out this limitation pertaining to sample diversity. We believe that the under-representation of rural residents in our sample could be related to the method of an online survey. Middle-aged Chinese residents who have access to the Internet and mobile devices are more likely to be urban residents with relatively privileged backgrounds. Therefore, the sample is not sufficiently diverse across urban and rural areas and the findings should not be generalized to the experiences of parents living in rural locations. We have added more detailed discussions relating to this limitation both in the Method section (on page 10) and the Limitation section (on page 28).

4. Relating to sampling, as far as I know, both WeChat and Little Read Book included more users who posed interests or fundamental knowledge on this topic, which may further confound selective reporting ingredients into this thematic coding. Please further justify this method over alternatives like interviews, especially considering the sensitivity of the topic.

RESPONSE: We agree that recruitment relying on social media platforms like WeChat and Little Red Book (now changed to Rednote according to the company’s update) may have led to sampling bias. This concern has been added in the Limitation section (on page 28). These two platforms were chosen due to their high popularity in China. The method of a qualitative survey was chosen mainly for ethical considerations. As mentioned in the Introduction section, some parents might view young people’s mental health difficulties as a sensitive topic, thus feeling shameful or uncomfortable discussing it with an outsider like the researchers. Especially, if participants’ children are personally experiencing mental health difficulties, they might feel it necessary to further conceal it in front of outsiders to preserve the family’s reputation. An online survey allowed them to answer all questions in a neutral and anonymous context, thus encouraging participants to talk more freely about their children’s conditions. Participants may also feel safer answering questions in this way instead of being interviewed face-to-face. Inevitably, a survey may not capture in-depth, detailed answers to questions as interviews. As requested by the reviewer, we have added additional information to both the rationale and limitations of this method in the Method section (on pages 9-10).

5. Coding Process. The coding process is described, but the manuscript would benefit from more detail on how inter-coder reliability was ensured, even if informal (e.g., through discussion and consensus-building between the authors).

RESPONSE: We have added more information to the methods section on pages 11-12 about the processes that were followed to build consistency in the application of the codes during the analysis process.

6. Age constraint. It is unclear why ages of their offspring are limited into 18-25. If in this case, they are all already adults. In the Chinese socioculture, such cohorts are no longer considered as representative for the entire population (e.g., 00 generation, 10 generation). Please justify this design and discuss potential biases introducing by this constraint.

RESPONSE: We appreciate the suggestion and we have added the missing rationale to the Method section (on page 9). The choice of this age range for children’s ages is based on developmental theories of emerging adulthood, which argue that the age span of 18-25 represents a crucial and sensitive life period when young adults experience various major life changes regarding identity, work, love and worldviews (Arnett, 2000). Research about global mental health also confirms that young adults aged 18-25 are particularly vulnerable to mental health risks due to unique challenges during this life period (WHO, 2020). Finally, as emerging adulthood represents a time when young people start gaining independence but are still partially dependent on their parents, we expect that it may bring different challenges and negotiations regarding healthcare decisions within the family context (Arnett, 2000), especially within the traditional hierarchal parent-child relationships in Chinese families, which have yet to be explored in the research literautre.

We agree with the reviewer that this design can limit the representativeness of the sample because it does not cover the entire population of the 00 generation. However, we do not aim for our study to represent the cohort, especially considering that this is a qualitative study with a relatively small sample of n=126 valid responses. Instead, our focus was on parents of emerging adults, and we aimed to understand detailed interrelationships between parents’ attitudes and children’s help-seeking for mental health during this life period. We have stressed this point now throughout the manuscript, including in the Limitations and future direction sections (on pages 28-29), stating that this study should not be interpreted as representative of cohorts or populations of the entire nation.

Arnett J. Emerging Adulthood: A Theory of Development from the Late Teens Through the Twenties. American Psychologist. 2000;55:469-480. doi: 10.1037//0003-066X.55.5.469.

World Health Organization. Suicide worldwide in 2019 [Internet]. www.who.int. 2021. Available from: https://www.who.int/publications/i/item/9789240026643.

7. Role of Gender. The gender dynamics in parental attitudes are not explicitly discussed. Given the cultural context, it would be interesting to analyze whether mothers and fathers exhibit different attitudes or coping strategies. In the Chinese cultures, societal expectation and roles largely vary from gender.

RESPONSE: We agree with the reviewer that social expectations of gender roles can vary considerably in traditional Chinese families. However, as this study was not designed to reflect gender differences in particular, the survey did not include specific questions to explore how mothers and fathers perceive their roles in the family respectively. Therefore, most answers are centred around similar key terms, with main differences focusing on causal attributions and attitudes towards young people’s help-seeking regarding mental health (for example, supportive or not). Our analyses did not point to meaningful gender differences regarding parents’ attitudes or support strategies. We have noted this in the discussion section and called for future research to further explore nuanced differences between mothers and fathers (see page 28).

Minor points

8. The manuscript would benefit from proofreading to correct minor grammatical issues and improve sentence flow.

RESPONSE: As noted in response to Reviewer 1’s point 6, we have revised the manuscript and addressed the grammatical errors in the previous version of the manuscript. We hope that the Reviewers will find that the quality of the writing has improved.

9. Terms like "mental health difficulties," "mental health issues," and "mental illness" are used interchangeably. It would be helpful to define these terms early in the manuscript and maintain consistency.

RESPONSE: We appreciate this suggestion. We have now revised the manuscript using the term “mental health difficulties (MHD)” as consistently as possible throughout the manuscript. We specify in the Introduction section that the term MHD will be used generally to refer to any below diagnostic-level symptoms, mental disorders, self-harm behaviours, suicide ideations and suicide attempts (see page 4). However, sometimes participants used terms such as “mental health issues”, “psychological problems”, and “mental illnesses” interchangeably within their answers. We believe that their choices of terminology may be based on and reflect their life experiences, perceptions, and understandings of MHD. Therefore, we decided to keep these terms in the quotations because we did not want to impose any terms on participants’ own words in the translation. Except for these quotations, MHD is now used consistently in other parts of the article, including the Introduction and the Discussion sections expect when key distinctions are needed (e.g., when referring to specific diagnoses of mental illness).
